# Congenital ileal-mesenteric fibrous band remnant of the vitelline artery causing small bowel obstruction in an adult female: a case report

**DOI:** 10.11604/pamj.2022.41.269.29624

**Published:** 2022-04-01

**Authors:** Prince Kasongo Mwila, Anchre Tersia Odendaal, Oula Ibrahim Ahmed, Baudouin Kongolo Kakudji

**Affiliations:** 1 Department of Surgery, Potchefstroom Hospital, Potchefstroom, South Africa,; 2 School of Clinical Medicine, Faculty of Health Sciences, University of the Witwatersrand, Johannesburg, South Africa

**Keywords:** Congenital, fibrous band, small bowel obstruction, adult, case report

## Abstract

The vitelline circulation connects the fetus and yolk sac in the first few weeks of fetal development. Its components are the vitelline duct, vitelline arteries and vitelline vein. This gradually breaks down as the placenta grows and overtakes the function of yolk sac as the primary nutrition source. In the event of persistence of these structures, multiple anatomical anomalies can arise such as a Meckel´s diverticulum, fibrous bands and others. We report on a rare finding of an ileal-mesenteric fibrous band remnant of a vitelline artery causing small bowel obstruction in an adult female. Our patient, a 40-year-old female presented with both clinical and radiological signs of small bowel obstruction. She had had no previous abdominal surgery or abdominal trauma. Intraoperatively we found an isolated ileal-mesenteric fibrous band situated at approximately 7 cm from the ileocecal junction. It spanned from the antimesenteric border of the terminal ileum to the border of the mesentery at a 15 cm breadth. In the snare-like loop that was created, part of the small bowel was trapped, creating a strangulated internal hernia which presented as a small bowel obstruction. The fibrous band was transected and the viable small bowel was freed during a laparotomy procedure. The post-operative period was uneventful and the patient was discharged on day 5. A fibrous band should be considered one of the rare causes of small bowel obstruction in a virgin abdomen in adults.

## Introduction

In the first few weeks of life, the essential source of nourishment to the developing fetus is the yolk sac. During the early stages this connection is via the vitelline circulation [1], which later breaks down gradually as the placenta grows and overtakes the role to become the primary nutritional source [1]. The vitelline circulation is composed of three anatomical structures: the omphalomesenteric duct, the vitelline arteries, and the vitelline vein. The omphalomesenteric duct is an embryonic tube that connects the two divisions of the primitive yolk sac, of which the larger one is the primitive gut and the smaller progresses as the yolk sac near the placenta [2]. The vitelline arteries pair off the aorta and pass on either side of the small intestine to supply the yolk sac. The vitelline veins traverse from the yolk sac to the sinus venosus. In the event that these embryonic structures are not obliterated completely, the following possible anatomical anomalies can arise: a fibrous band (FB), Meckel´s diverticulum (MD), a mesodiverticular band, an entero-umbilical fistula, an umbilical sinus, enterocystoma of the ileum, and congenital umbilical abnormalities [3].

Although a MD in its anti-mesenteric position is the most common malformation of the omphalomesenteric duct, the aforementioned abnormalities have been reported in literature. This has furthermore been reported even with the MD located at the mesenteric edge [4]. The aim of this paper is to report on a rare intraoperative finding of an ileal-mesenteric FB remnant of a vitelline artery causing small bowel obstruction in an adult female.

## Patient and observation

**Presenting complaint and past history:** a 40-year-old female presented to our emergency department as a referral from a local clinic. She had a 4-day history of obstipation, a 3-day history of vomiting with associated loss of appetite and generalized colicky abdominal pain. The patient had no previous abdominal surgery or significant medical history.

**Physical examination and clinical findings:** on physical examination she was acutely ill-looking, with the following vitals: blood pressure of 104/68 mmHg, pulse rate of 105 beats per minute, respiratory rate of 20 breaths per minute, saturation of 95% on room air, and temperature of 36.2°C. Her abdomen was found to be mildly distended, hyperesonant on percussion, with voluntary guarding to palpation that was most severe in the epigastrium, and increased bowel sounds on auscultation. Notably, no signs of peritoneal irritation were present. Digital rectal examination revealed she had soft stools high in the rectum and no palpable mass. A urine pregnancy test was negative.

**Figure 1:**
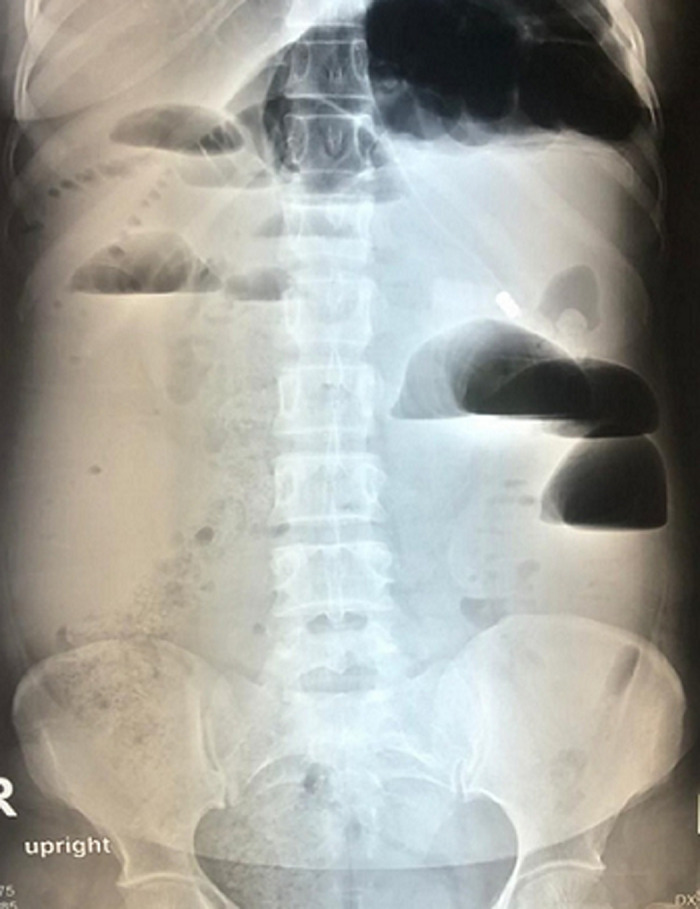
multiple air fluid levels

**Imaging and blood tests:** radiological investigations included an erect chest X-ray and erect and supine abdominal X-rays. They demonstrated dilated small bowel loops and multiple air-fluid levels with no free air under the diaphragm (Figure 1). Laboratory investigations yielded an unremarkable full blood count and the following deranged electrolytes: hyponatraemia of 125 mmol/L, hypokalaemia of 3.3 mmol/L and hypochloraemia of 84 mmol/L. The patient was mildly dehydrated with a urea of 10.5 mmol/L and creatinine of 96 umol/L. The arterial blood gas done demonstrated a metabolic alkalosis with a pH of 7.50, lactate 1.3, HCO^-3^28.1 and BE 5.0.

**Figure 2:**
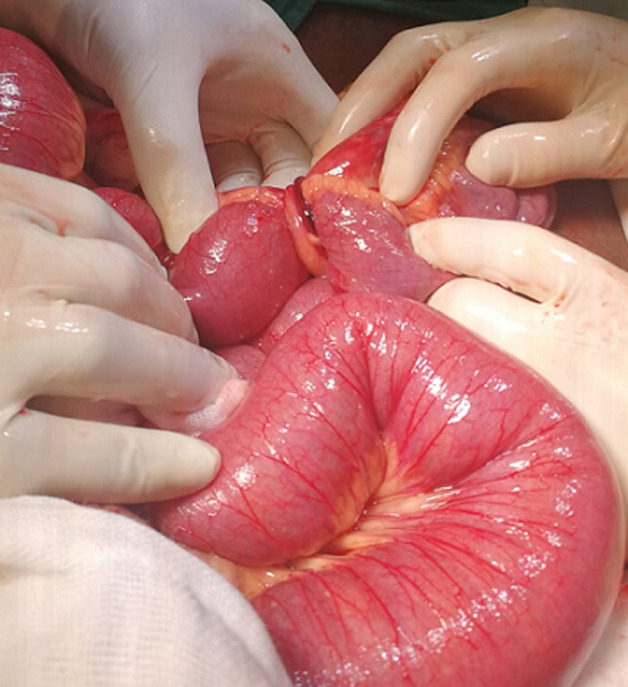
fibrous band encircling small bowels

**Figure 3:**
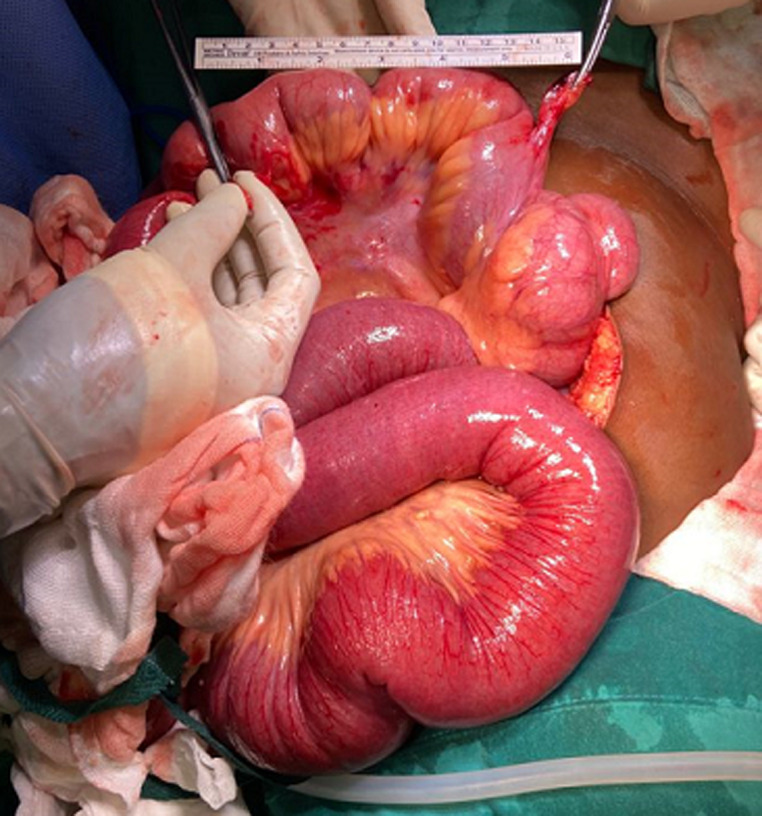
proximal and distal attachment of the fibrous band and distance in between

**Figure 4:**
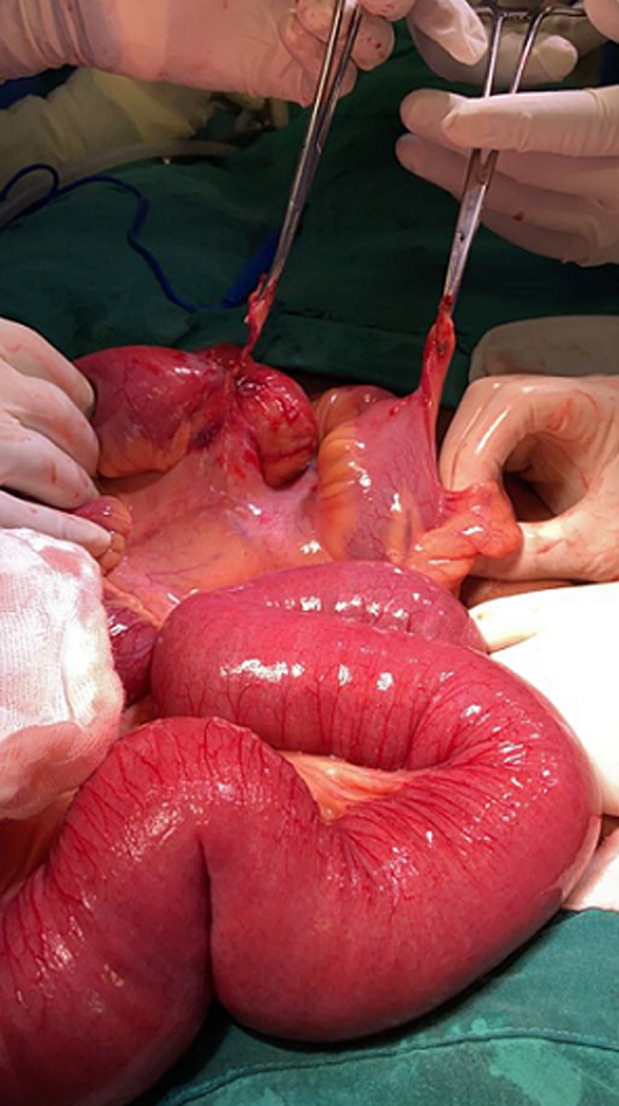
transected fibrous band with viable bowel

**Assessment and therapeutic intervention:** an assessment of mechanical bowel obstruction was made. The patient was optimized with one liter of normal saline. A nasogastric tube that was inserted drained approximately two liters of bilious content, a urinary catheter was also inserted during the optimization for surgery. Intraoperatively, dilated loops of bowel were found proximally to an isolated fibrous band encircling the small bowel (Figure 2). The distal part of the band, situated 7 cm from the ileocecal junction was attached to the antimesenteric edge of the ileum 15 cm away from the proximal part, which in turn was attached to the mesenteric side of the ileum (Figure 3). The fibrous band was transected between two artery forceps. The small bowel entrapped within the fibrous band was inspected and found to be viable (Figure 4).

**Follow-up and outcomes:** the immediate post-operative recovery period was uneventful and the patient was discharged on day 5. The histology report showed a fibro-fatty connective band with moderate vascular congestion, areas of fibrosis, fresh hemorrhage and granulation tissue. The patient had made a full and uncomplicated recovery by the time of her last follow-up visit two months later. She was satisfied with the management and the outcome.

## Discussion

A 40-year-old female presented with clinical and radiological signs of small bowel obstruction. We found an isolated ileal-mesenteric FB intraoperatively, situated at approximately 7 cm from the ileocecal junction. It spanned from the antimesenteric border of the terminal ileum to its mesenteric side about 15 cm proximally. In the snare-like loop that was created, part of the small bowel was trapped, creating a strangulated internal hernia which led to the small bowel obstruction. Literature shows that a considerable number of bowel obstructions are caused by abnormalities from the omphalomesenteric duct. This results from various mechanisms. Some of the common mechanisms include Intussusception (where the MD acts as the leading point); volvulus (caused by the small intestine twisting around a fibrous band from the MD to the anterior abdominal wall); and internal herniation (caused by a band attached to another viscus) [5,6].

Very few articles report small bowel obstruction caused by an isolated fibrous band at the ileal mesenteric location in an adult as illustrated in this case report. Furthermore, of those reported, most are found in the pediatric population [5]. A recent publication by Kerkeni *et al*. anomalous bands (CAB) into two categories [7]. The first group is CAB with definable origins, namely remnants of embryological structures, bands related to intestinal rotation, and fixation anomalies and bands encountered in meconium peritonitis. The second group is classified as idiopathic congenital bands for which a clear explanation of the origin is lacking [7]. CABs are common in the pediatric population where they can cause different clinical presentations ranging from intermittent abdominal pain to bowel obstruction. However, only a few cases have been reported in adults [8,9].

This case is comparable to a mesodiverticular band considered to have originated from a vitelline artery. This has also been reported by other authors to be a cause of bowel obstruction [2,10]. For Omer *et al*. the mesodiverticular band may or may not be associated with an MD [1]. The absence of an attachment of the band to a MD in our case intrigued us in terms of the real nature of the ileal-mesenteric band we found in that it can arguably not be called a mesodiverticular band because of the absence of the MD attached to it. A finding of a single FB stretching from the antimesenteric border of the terminal ileum to the mesenteric edge of the ileum 15 cm apart suggests that it is a remnant of a vitelline artery and not the vitelline duct. In support of this, other authors have also reported an isolated band originating at the terminal ileum without a MD, causing a bowel obstruction [9,10].

**Strength and limitation:** if diagnosed earlier, management is relatively simple and consists of splitting the FB with the release of the entrapped bowel. There was no delay in the diagnosis and the surgical decision to operate. This could be considered as strength of this case presentation. In the event of a delayed diagnosis, a bowel resection with primary anastomosis might have been needed, regardless of the age of the patient. The limitation was the lack of a functional laparoscopic unit as the patient could have benefited from a laparoscopic approach.

## Conclusion

We have presented the case of a 40-year-old female with bowel obstruction secondary to an entrapped small bowel in a fibrous band remnant of the vitelline artery. The FB spanned from the antimesenteric border of the terminal ileum to the mesenteric edge of the ileum at a breadth of 15 cm. The FB was transected and the post-op period was uneventful. FBs are a rare yet possible cause of small bowel obstruction in an adult with a virgin abdomen.
